# Neurons Controlling Voluntary Vocalization in the Macaque Ventral Premotor Cortex

**DOI:** 10.1371/journal.pone.0026822

**Published:** 2011-11-02

**Authors:** Gino Coudé, Pier Francesco Ferrari, Francesca Rodà, Monica Maranesi, Eleonora Borelli, Vania Veroni, Fabio Monti, Stefano Rozzi, Leonardo Fogassi

**Affiliations:** 1 Dipartimento di Neuroscienze, Università di Parma, Parma, Italy; 2 Dipartimento di Biologia Evolutiva e Funzionale, Università di Parma, Parma, Italy; 3 Dipartimento di Psicologia, Università di Parma, Parma, Italy; 4 Istituto Italiano di Tecnologia, Rete Multidisciplinare Tecnologica, Parma, Italy; University of Minnesota, United States of America

## Abstract

The voluntary control of phonation is a crucial achievement in the evolution of speech. In humans, ventral premotor cortex (PMv) and Broca's area are known to be involved in voluntary phonation. In contrast, no neurophysiological data are available about the role of the oro-facial sector of nonhuman primates PMv in this function. In order to address this issue, we recorded PMv neurons from two monkeys trained to emit coo-calls. Results showed that a population of motor neurons specifically fire during vocalization. About two thirds of them discharged before sound onset, while the remaining were time-locked with it. The response of vocalization-selective neurons was present only during conditioned (voluntary) but not spontaneous (emotional) sound emission. These data suggest that the control of vocal production exerted by PMv neurons constitutes a newly emerging property in the monkey lineage, shedding light on the evolution of phonation-based communication from a nonhuman primate species.

## Introduction

Nonhuman primates vocalize in a wide range of contexts and possess a repertoire of vocalizations used to designate objects, events or affective states [1], [2]. The extent to which this vocal behavior is under voluntary control remains controversial. Some species are capable of modifying their vocalizations according to environmental parameters [3], [4], [5], however, none of them is granted with enough flexibility to learn completely new vocal patterns (see [6]). Due to this apparent lack of flexibility, nonhuman primate vocal behavior was traditionally assumed to be predominantly emotional [7], [8], [9], and consisting chiefly in a repertoire of involuntary or reflexive responses to a range of specific valence stimuli [10], [11]. Partly conflicting with this view, behavioral studies showed that macaques can have a limited control on vocalization. Several investigations, in fact, demonstrated that they can achieve a significant level of voluntary vocal control when submitted to operant conditioning tasks [6], [12], [13], [14], even though the success rate obtained in those studies is highly variable [15], [16].

Interestingly, the neural underpinnings of vocal production in nonhuman primates have been classically attributed to the brainstem and to areas of the mesial cortex that, besides other functions, are also involved in emotional behavior [17], [18], [19]. Electrical stimulation experiments showed that anterior cingulate gyrus yields species-specific vocalizations in squirrel monkeys [20] and in macaques [21]. Further experiments involving brain lesions and single-unit recordings provided additional evidence of the key role of mesial cortex in vocalization [17].

In addition to these regions, a potential candidate cortical region for voluntary control of vocalization is the ventral premotor cortex (PMv). This region is well known to control hand and mouth actions [22] and contains a larynx movements representation, as shown by electrical stimulation studies [19], [23]. Furthermore, part of it is considered homologue of human Broca's area [24], [25], which is involved in speech production [26], [27], [28], [29] and larynx control [30]. Although monkey PMv has been considered to have a minor involvement in vocalization [19], [31], [32], the properties of its neurons in this function have not been directly investigated.

The aim of this study was to verify whether neurons in macaque area PMv are directly involved in conditioned vocalization. We addressed this issue by training macaque monkeys to produce conditioned vocalization, and then recording single neurons from the lateral sector of PMv during voluntary-controlled and spontaneous vocalizations. Finally, we verified by electrical microstimulation whether the recording regions have a motor output on the mouth and larynx.

## Materials and Methods

Two captive-born and individually housed adult pigtailed macaques (*Macaca nemestrina*) served as subjects. All experimental protocols were approved by the Ethical Committee for Animal Research of the University of Parma and by the Superior Institute for Health (last appraisal no. 2783, 26/01/2010). The authorization for conducting our experiments was delivered by the Animal Health and Veterinary Medication Division of the Department of Public Veterinary Health, Nutrition and Food Safety of the Italian Ministry of Health (permit by ministerial decree no. 6/99-A, 29/01/1999; last renewals. no. 54/2010-B, 55/2010-C, 18/03/2010). The monkeys were housed and handled in strict accordance with the recommendations of the Weatherall Report about good animal practice. Our routine laboratory procedures included an environmental enrichment program where monkeys had access to toys, mirrors and swings. They also had visual, auditory and olfactory contact with other animals and, when appropriate, could touch/groom each other. Any possible pain associated with surgeries was pharmacologically ameliorated. The well-being and health conditions of the monkeys were constantly monitored by the institutional veterinary doctor of the University of Parma.

**Figure 1:**
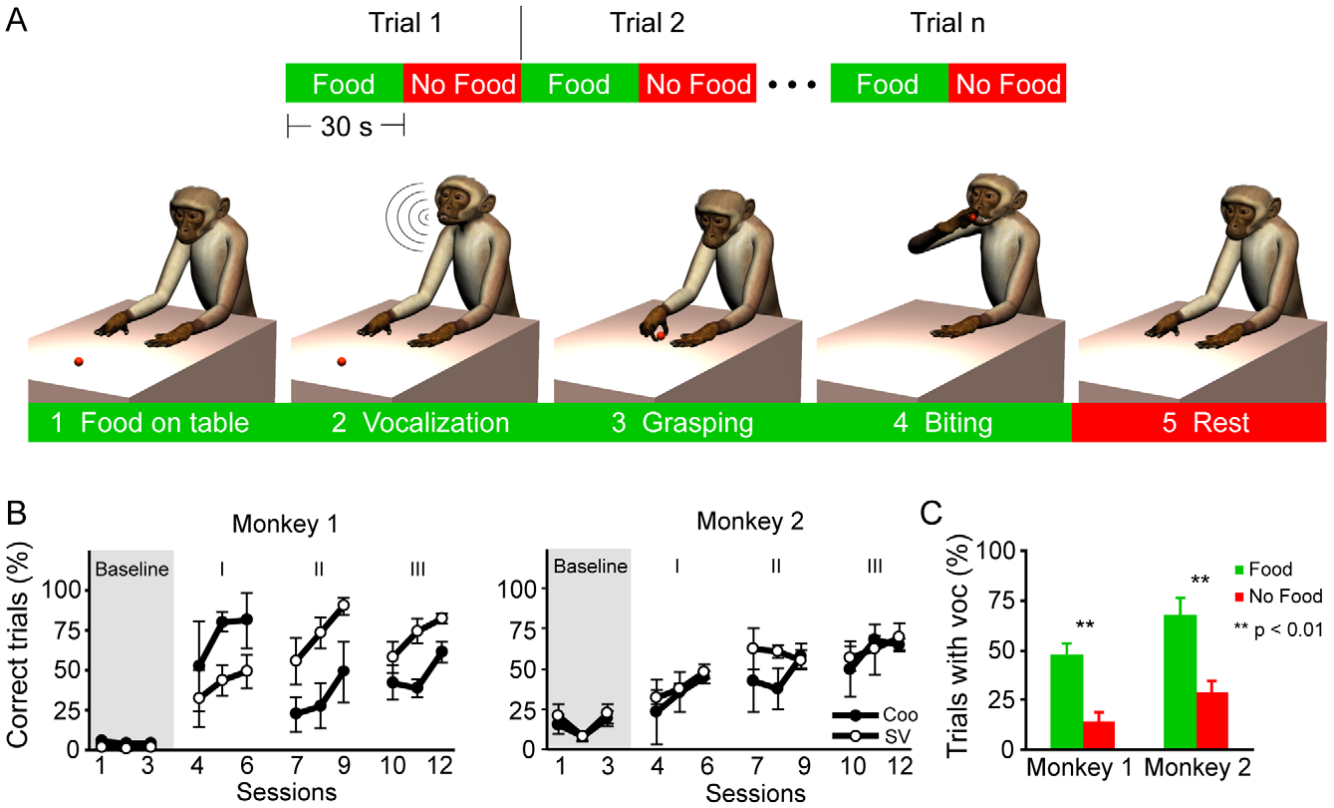
Vocal operant conditioning task. **A.** *Top.* Each trial consisted in a “Food” (green) and a “No Food” (red) condition. *Bottom.* Schematic illustration of a successful trial of the task. In the “Food” condition the food was presented on a table (1); if the monkey emitted a coo (2), the food was given to the monkey that grasped it (3) and brought it to the mouth (4). In the “No Food” condition the food was not present and the monkey was not required to vocalize (5). **B.** Monkeys performance throughout baseline and training phases. Each dot of the line graph represents the average percentage of the performance on three consecutive training sessions. For the sake of clarity only the sessions taken from the onset, middle and end of each training phase are shown. Black dots = successful vocalization trials; empty dots = vocalization trials in which SV occurred. **C.** Percentage of trials with vocalization in “Food” and “No Food” conditions for the two monkeys. Error bars represent s.e.m.

Both monkeys were chosen because they displayed a higher level of spontaneous coo-call emission with respect to their peers housed in the lab facility. Before starting training, a baseline level of spontaneous coo-call (frequency of calls/session) was assessed. The monkeys were then submitted to an operant conditioning task designed to increase their vocalization rate. The task initially consisted of a shaping procedure, lasting 5 days, in which the monkeys were rewarded by the experimenter with a piece of palatable food any time they emitted a coo-call. At the end of this procedure, the following structured task was introduced: (a) The monkeys were facing a small table and had to emit a coo-call only when a piece of food, kept at an out-of-reach distance, was put on the table by the experimenter (Fig. 1A); (b) The monkeys had to emit a vocalization within the 30 s duration of the presence of food on the table (“Food” condition) in order to get it as a reward; if they did not vocalize within 30 s, food was removed from the table. (c) At the end of the “Food” condition period, a “No Food” condition (30 s duration) followed, where no food was present on the table and no reward was delivered to the monkeys, even in the case they vocalized. Each session of the task was 30 minutes long. Since the task was very sensitive to monkey fatigue, a maximum of two sessions/day were carried out.

The task was performed in three subsequent phases aimed at gradually training the monkey to perform the task during the electrophysiological recording. Each new phase involved a change in the environmental conditions. First, the task was performed when the monkey was in its home cage (Stage I). Second, the monkey performed the task while seated in the primate chair with the head free (laboratory training, head free – Stage II). Third, the task was performed while the monkey was seated on the primate chair with the head fixed by a head holder designed for electrophysiological recordings (laboratory training with the head fixed – Stage III). During the task, other types of vocalization like grunts or shrieks were never reinforced. Throughout the whole training, the behavior of the monkeys was carefully recorded on a data sheet, especially the frequency with which vocalizations were produced. We also recorded the occurrence of a specific orofacial configuration that normally accompanies coo-call execution, but that is made without sound production. This behavior was observed in a previous lesion study [31] and described as “silent vocalization” (SV) because the facial display is very similar to that occurring during vocalization.

### Surgical procedure

As vocalization training with the head free was deemed complete, each monkey underwent surgery. The surgical procedures for single-unit recordings were the same as previously described [33], [34]. Each animal was deeply anaesthetized with ketamine hydrochloride (5 mg/kg i.m.) and medetomidine hydrochloride (0.1 mg/kg i.m.) and its heart rate, temperature and respiration were carefully monitored and kept within physiological range. Pain medication was routinely given after surgery. The head implant included a head holder and a custom-made titanium chamber for single-unit recordings implanted stereotaxically. In the two animals, the size of the recording chamber in its rostro-caudal and medio-lateral axes (about 20 mm×25 mm) was such as to allow recording from the whole ventral premotor cortex, including also area F1 (primary motor cortex) and the caudal part of the frontal eye fields (FEF).

### Electrophysiological recording and microstimulation

Neurons were recorded using glass-coated tungsten microelectrodes (impedance 0.5–1.0 MΩ, measured at 1 kHz) inserted through the intact dura. Neuronal activity was amplified, monitored on an oscilloscope and played through an audio amplifier. The monkey calls were recorded by means of a high definition microphone (Earthworks TC30) placed near the monkey. Both neuronal and voice signals were digitalized at 44.1 kHz through an analog/digital interface (Mindprint AN/DI pro) and sent to a PC where they were displayed online and saved for offline analysis. Individual action potentials were isolated with a dual voltage-time window discriminator (Bak Electronics, Germantown MD, USA). The output signal from the voltage-time discriminator was monitored and fed to a PC for analysis. All recording sessions were video-recorded. Intracortical microstimulation was also carried out with the same microelectrodes used for recordings (train duration: 50 ms; pulse duration: 0.2 ms; frequency: 330 Hz; current intensity: 3–40 µA). When recording sites showed neuronal activity related to vocalization, longer stimulation train were also used (train duration: 500–1000 ms; pulse duration: 0.2 ms; frequency: 50–200 Hz; current intensity: 30–60 µA).

### Neuronal testing and functional mapping

After chamber implantation, the ventral part of the agranular frontal cortex was functionally explored (single-unit recordings and intracortical microstimulation) in order to assess the location of areas F1 (primary motor cortex), F4 and F5 (PMv) and to find out the sector of F5 where neurons related to mouth actions are mostly located [33]. The criteria used to functionally characterize the different areas were the same as used by Fogassi et al. [35]. *Area F1*: low threshold of excitability to microstimulation, discharge during active movements, response to passive somatosensory stimuli. *Area F4*: moving the electrode rostrally from F1 hand field, appearance of proximal and axial movements to electrical stimulation, increase in stimulation threshold, appearance of visual responses, presence of large tactile receptive fields located on the face and body and of visual peripersonal receptive fields around the tactile ones. *Area F5*: going further rostrally, re-appearance of distal movements though requiring higher stimulation currents than F1, disappearance of spatially organized receptive field typical of F4, presence of visual responses to the presentation of 3D objects or complex actions, presence of a large number of neurons discharging in association with goal-directed movements. The recorded area was sampled with a 1 mm grid. A total of 205 electrode penetrations were carried out in the lateral parts of areas F4 and F5 of the two monkeys. Overall, vocalization-related neurons were found in 7 electrode penetrations, all of them located in the rostral half of the recorded region. Sectors in which activity related to vocalization was found were subsequently more densely sampled. Note that the sites in which vocalization-related neurons were found contained also neurons coding different mouth- or hand-related motor acts.

The neurons were studied in more details as follows: once the first neuronal activity was found, the neurons were “clinically” tested to establish their motor, somatosensory, visual or auditory properties. This testing consisted in assessing neuron properties in semi-naturalistic situations in which the monkey was allowed to reach, grasp, lick, bite, smell food or objects and to interact with the experimenter. If motor properties related to mouth were found, the vocalization task described above started, otherwise the electrode was slowly lowered in steps of 250 µm of depth. When an action potential was isolated, we tested the unit for vocalization as well as for other behaviors related to mouth/throat movements like mouth grasping, licking, swallowing or chewing. Somatosensory stimuli for limbs, trunk and face consisted in bending hairs, touching the skin, applying light pressure to the tissue, and slow and fast rotations to the joints. Light pressure on the muscle belly and tendons were also applied. Oral cavity tactile stimuli were applied using a small stick or spatula. All testing was performed with monkey's eyes open and closed.

zBesides clinical testing, auditory properties have been investigated more in depth in order to evaluate whether vocalization-related neurons could be also activated when the monkeys were listening to conspecifics vocalizations (mirror responses). To this purpose, we played back the vocal emissions of the tested monkeys and other vocalizations recorded from several monkeys of our facilities. We also recorded on-line the monkey vocalization and immediately played back the same vocalization that was effective during monkey sound emission. These procedures enabled us to rule out the possibility that the neuronal discharge during vocalization was due to simple auditory feedbacks. The same software used for recording vocalization was employed for sounds play-back. All acoustic stimuli were presented using a single high-quality digital loudspeaker (Genelec S30D) having a frequency response reaching 48 kHz (with an amplitude variation within ±2.5 dB range), placed 2 m from of the monkey.

Intracortical microstimulation trains were applied during each penetration every 500 µm after having tested the neuronal properties. The stimulation was delivered only when the monkey was relaxed and remained still for a few seconds. A light-emitting diode and a soft sound were instantly turned on during stimulation and enabled us to assess the temporal coincidence between stimulation trains and motor output. Oro-facial and brachio-manual movements were visually detected by two experimenters, while larynx movements were assessed by tactile probing, i.e. the experimenter gently applied his/her fingers over on the throat skin covering the larynx.

### Off-line data analysis of neuronal trace

For each recorded site, the time occurrence of behavioral events of interest (e.g. vocalization, biting, licking, mouthing, hand grasping, etc.) was precisely identified by means of the video recordings synchronized with neuronal activity trace. The neuronal activity trace associated with the various behaviors was submitted to a spike sorting analysis performed with a Matlab-based program (wave_clus, Caltech). We acquired the activity of each neuron for several trials during the monkey performance of different behaviors.

For each behavior, neuronal discharge was aligned with specific events: (a) vocalization, with the onset of sound emission; (b) mouth grasping, with the contact between mouth and food; (c) licking, with the contact between tongue and food, (d) chewing, with the first maximal aperture of the mouth, (e) hand grasping, with the contact between the hand and the food. This procedure allowed to construct rasters and PSTH and to subsequently compare the neuronal discharge recorded during monkey performance of different behaviors.

The analysis of neuronal activity for each behavior was carried out by considering the discharge of two epochs having a duration of 400 ms each. The choice of this timing is based on an analysis which revealed that the duration of the relevant behaviors is within a range of 350–450 ms. The epochs were identified according to criteria that were adapted to the specific behavioral features. *Vocalization*. Epoch 1: from the beginning of lips opening before vocalization to sound emission onset; epoch 2: from sound emission onset to the end of lips protrusion. *Licking, mouth grasping and chewing*. Epoch 1: from beginning of mouth opening to contact with food; epoch 2: from contact with food to mouth closure. *Hand grasping*. Epoch 1: from beginning of hand opening to contact with food; epoch 2: from contact with food to hand closure. For the baseline condition (rest), we considered an 800 ms period during which the monkeys were not performing any mouth or hand movement. In each trial, the mean discharge frequency was calculated for each epoch.

### Statistical analysis

#### Behavioral and single neuron analysis

For the behavioral analysis, the monkey performance was calculated in terms of number of trials/session in which at least one vocalization or SV was emitted during the “Food” or “No Food” condition. The Wilcoxon matched pairs test was used to assess for significant difference in the frequency of vocalization between training phases and between conditions (“Food” or “No Food”). The neurophysiological data on single neurons were analysed with a two-way ANOVA for repeated measures (alpha: p<0.05) (factors: Epoch and Condition) followed by a Newman–Keuls test (two-tail, p<0.05).

#### PMv raw activity measure and population analysis

To compare neuronal activity associated with the emission of conditioned (“Food”) and spontaneous calls (“No Food”), we performed a population analysis, by applying a method that calculates neuronal activity using the RMS estimate of the raw multi-unit activity [36]. The RMS estimate is defined as follows:
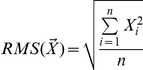
Where 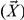 is the vector of the analog signal trace, 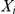 is each sample squared (a sample consisted in the raw neuronal activity trace for a single trial), and 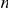 is the number of samples. Since RMS is influenced by electrode properties, amplification gain, or other factors, the RMS was normalized (NRMS) on the baseline activity recorded when the monkey did not produce any movement (baseline NRMS activity = 1). The bin width of the RMS is 20 ms, the same as in single-unit PSTH analysis.

Normalized RMS data were submitted to an arcsin-square root transformation. A 2×2 repeated measure ANOVA (Factors: Condition and Epoch) has been applied to the NRMS for “Food” and “No Food” conditions in the two epochs before (Epoch 1) and during (Epoch 2) the vocalizations.

### Voice recordings and analysis

All sounds recorded during the experiments were digitalized at 16 bits quantization and 44.1 kHz sampling frequency. Each vocalization was trimmed from other sounds and band-pass filtered from 30 Hz to 10 kHz by means of a sound editing software (Cool Edit/Audition, Adobe) to remove hissing and subsonic rumble. All voice analysis was performed with Praat analysis software package. The differences in acoustic measures between “Food” and “No Food” conditions were analyzed with paired t-test (two-tail, p<0.05).

### Histological procedures and data analysis

About 10 days before sacrificing the animals, electrolytic lesions (10 µA cathodic pulses for 10 s) were performed at known coordinates at the external borders of the recorded regions. Each animal was then anaesthetized and transcardially perfused as previously described [34]. The brain was frozen and cut in 60 µm-thick sections, with each second and fifth section of a series of five stained using the Nissl method (thionin, 0.1% in 0.1 M acetate buffer pH 3.7). The locations of penetrations were then reconstructed on the basis of electrolytic lesions, stereotaxic coordinates, depths of penetrations and functional properties.

## Results

### Behavioral performance in vocalization

The vocal operant conditioning task consisted of 115 sessions in Monkey 1 and of 109 sessions in Monkey 2 (Figure 1A). The overall progress of the two monkeys performance throughout the various phases of training is shown in Figure 1B. As expected, before training, the frequency of vocalization (filled circles) was low for both monkeys as indicated by the baseline mean percent of successful trials (Monkey 1, 4.4±1.0%; Monkey 2, 14.1±2.9%). The overall vocal performance increased with training, reaching a final value of 61.1±6.8% in Monkey 1 and 64.4±4.0% in Monkey 2 at the end of training.

Since the earliest signs of progression in vocal learning, we noticed that trained vocalization required considerable efforts in both animals. Indeed, they often failed in emitting any sound, while still producing the characteristic lips configuration that normally accompanies coo-call execution (SV – see Figure S1 and compare vocalization in Movie S1 to SV in Movie S2). Interestingly, the percentage of SV (empty circles) made during the baseline phase was nearly zero. As training proceeded, the number of SV rose, together with the number of actual vocalizations, suggesting that this behavior is a failed attempt to vocalize. This idea is in line with the fact that this gesture does not correspond to any other known pig-tail macaque communicative facial expression and that it was never observed in untrained monkeys housed in our facility.

In some occasions, failed vocalizations took an alternative form and were not completely silent, but rather consisted in an audible air sound, toneless, and similar to a whisper. We hereafter refer to that vocal production as “air blow”.

### Neuronal responses during vocalization

**Figure 2:**
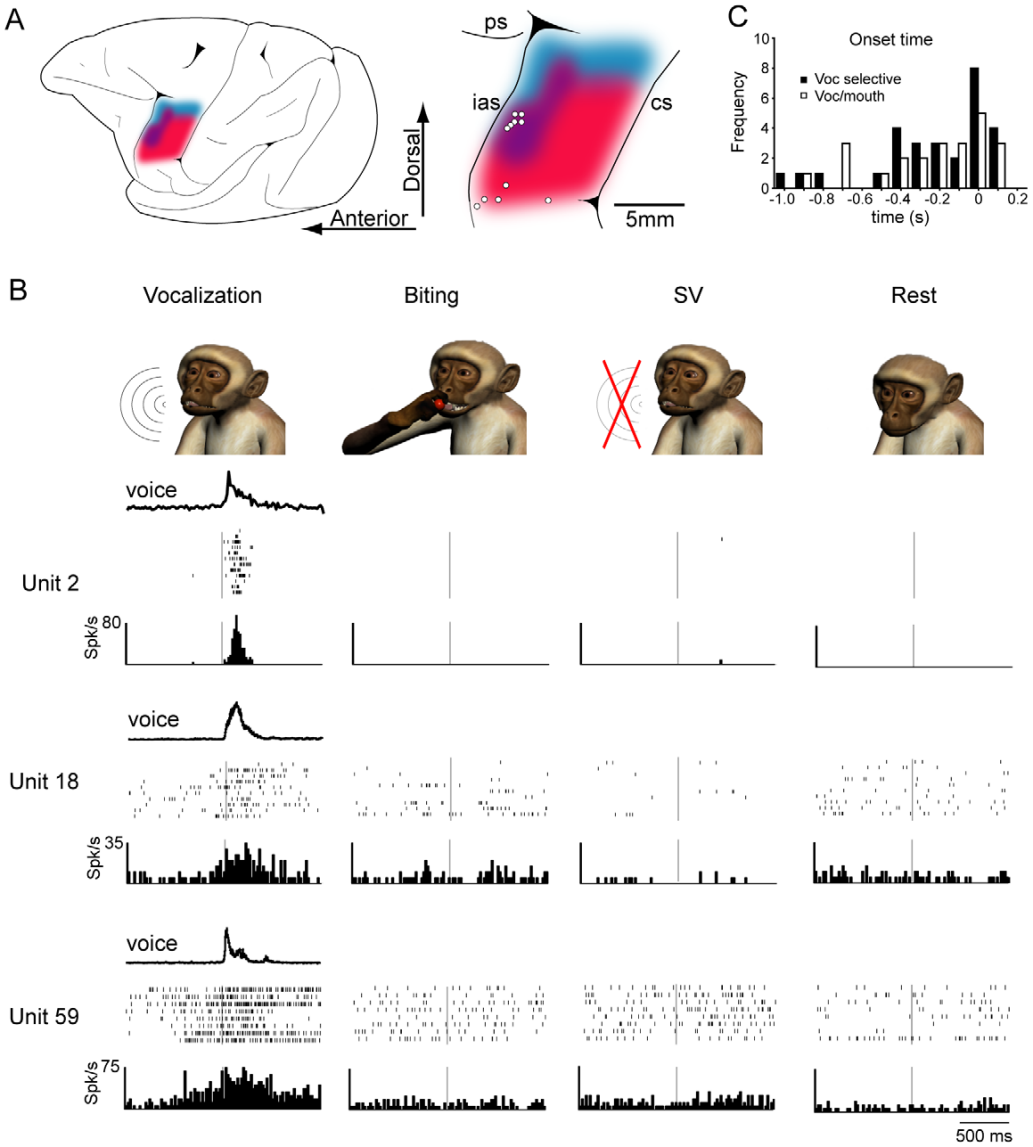
Recorded region and vocalization-selective neurons. **A.** *Left.* Lateral view of the left hemisphere of Monkey 2. Colored sectors indicate hand (blue), mouth (red) and overlapping hand and mouth (purple) motor representations. *Right.* Enlarged view of the recorded area showing the position of electrode penetrations (white dots) where vocalization-selective neurons were found. Note that some penetrations overlapped. cs = central sulcus, ias = inferior arcuate sulcus, ps = principal sulcus. **B.** Examples of three vocalization-selective neurons recorded during four different behaviors. For each unit, rasters and histograms illustrate the neuronal discharge aligned (vertical gray line) with behavioral events. They correspond to monkey sound emission onset during vocalization, contact with food during biting and maximum lips protrusion during silent vocalization (SV). During rest, the activity alignment corresponded to the midpoint of a period in which the monkey did not produce any movement. The root mean square of sound trace of monkey vocalization is depicted for each unit above the response raster related to vocalization. **C.** Frequency of vocalization-selective (black bars) and vocalization/mouth related neurons (white bars) according to discharge time onset with respect to the beginning of sound emission.

Once the vocalization rate reached a criterion of 60%, a functional characterization of the PMv neuronal properties was performed in both monkeys (see Methods). Figure 2A (left) shows the PMv sectors containing neurons related to hand, mouth, or hand and mouth representation. During this investigation we also identified sectors in which neurons correlated to vocalization could be found. A total of 106 neurons in 31 penetrations have been studied to assess whether the discharge was correlated with vocalization. Out of them, 63 neurons discharged in relation to vocalization. In fifty-one of them we recorded a sufficient number of trials allowing statistical analysis. All these neurons presented a significantly higher discharge during vocalization with respect to rest condition. Twenty-eight showed a selective discharge for vocalization (see Table 1) when compared to other behaviors (see Materials and Methods). The location of penetrations of Monkey 1 in which vocalization-selective neurons were found is given in Figure 2A (right). Their location appears to include the lateral part of area F5 and, possibly, expands ventrally to it, in dorsal opercular area (DO; for a characterization of the different sectors of area F5, see [37]). Figure 2B shows three examples of this type of neurons. Note that no discharge was present during other types of mouth-related behavior (Figure S2). Neuronal discharge was time-locked with the onset of sound emission in Unit 2, while preceded vocalization in the other two units. The distribution of firing onset of all vocalization-related neurons is shown in Figure 2C. In most recorded vocalization neurons the discharge started before sound onset (71% of vocalization-selective and 65% of vocalization/mouth-related). The discharge of vocalization-selective neurons does not depend on auditory feedback, since we never recorded responses when the vocalizations were played back (see Material and Methods). Furthermore, tactile stimulation applied inside the mouth, on the tongue, face and anterior neck did not elicit any response, showing that also somatosensory feedback cannot justify the neural discharge.

**Table 1:** Number of neurons responding during trained vocalization.

	Before Sound Onset	During Sound Emission	All
**Vocalization-selective**	20	8	28
**Vocalization/mouth related**	16	7	23
***Total***	*36*	*15*	*51*

**Figure 3:**
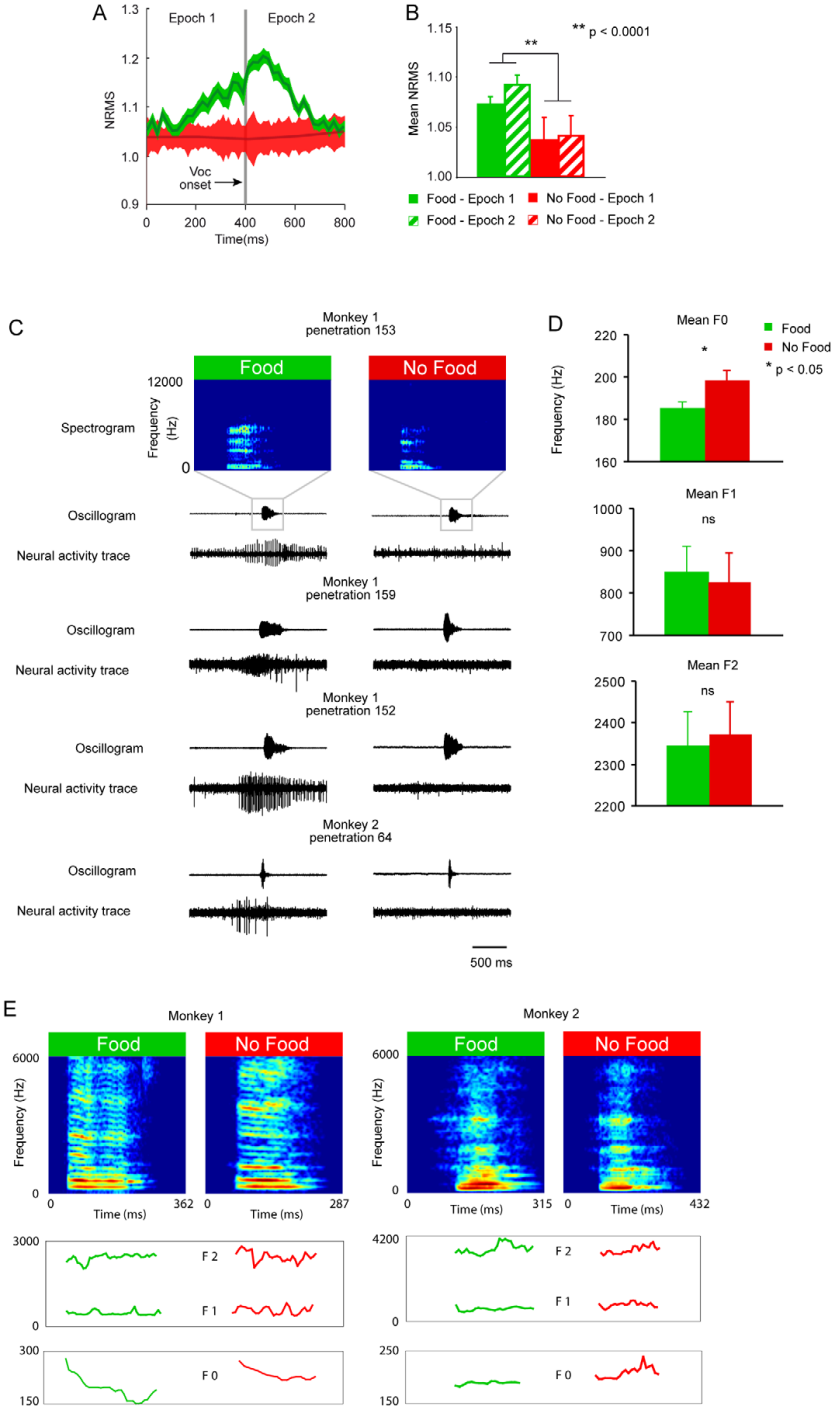
Vocalization neurons specificity for “Food” condition. **A.** Time course of the population of NRMS of neuronal activity associated with “Food” (n = 320) and “No Food” (n = 37) vocalizations. The mean NRMS of “Food” and “No Food” conditions are represented by the dark green and dark red lines, respectively. The light shaded colors represent ±1 SEM. **B.** Histograms (means ± SEM) showing the comparison between “Food” and “No Food” NRMS calculated in the epochs before sound emission (Epoch 1) and during and after sound emission (Epoch 2). **C.** Neuronal activity and sound features of coo-calls produced during “Food” and “No Food” conditions. Examples of different neurons firing only during “Food” vocalizations. **D.** Comparison of mean F0, F1 and F2 of coos made during “Food” and “No Food” conditions. Only F0 bears significant differences between the two conditions (paired t-test. P<0.05). **E.** Examples of sonograms of coos made during “Food” and “No Food” conditions. The differences in F0 and the similarities in F1 and F2 are illustrated in the boxes below the sonograms. In the x-axis time scale is the same of the sonograms depicted above.

It was also verified whether vocalization-related neuronal activity was present during spontaneous vocalization (“No Food” condition). This is a difficult issue to address since spontaneous vocalizations were very few after the training period (Figure 1C). Therefore, the comparison of single-units recorded during both “Food” and “No Food” vocalization would yield only few cases. To overcome this limitation, we performed a population analysis in which we compared the normalized root mean squared (NRMS) of the raw neuronal activity trace during all coo-calls made by the two monkeys in the “Food” and “No Food” conditions (n = 320 and 37, respectively; see Materials and Methods for details). Figure 3A shows the time course of the mean NRMS neuronal activity for the two conditions. Only “Food” condition presents a rise of activity associated with the call emission. A 2×2 repeated measure ANOVA (Figure 3B) showed a significant Condition×Epoch interaction (F_2,35_ = 10.83, *p*<0.0005). In particular, neuronal activity associated with the coos made in the “Food” condition is significantly different from that recorded during the “No Food” (p<0.0005).

In addition to population analysis, the neuronal specificity for “Food” vocalization was also directly assessed in nine neurons in which it was possible to directly compare the activity of the same unit in both “Food” and “No Food” conditions. The results showed that the discharge was present only for vocalizations emitted in the “Food” condition (Figure 3C). In order to understand whether this difference in discharge is related to a difference in sound emission, we investigated sound characteristics in the two basic conditions of the task, by measuring the fundamental frequency (F0) and the first (F1) and second (F2) formants of the vocalizations. Figure 3D shows the mean F0, F1 and F2 measured for 30 coos for each condition in the two monkeys. The two conditions are significantly different in the F0 component (Z = −2.24, *p*<0.05, two-tailed Wilcoxon paired-sample test) but not in F1 (Z = 0.53, ns) and F2 (Z = −0.55, ns), indicating that the differences in sound emissions between conditioned and non-conditioned vocalizations imply different larynx movements but similar mouth articulation. Examples of the spectrograms of coo-calls recorded during individual trials in the “Food” and “No Food” conditions are shown in Figure 3E.

**Figure 4:**
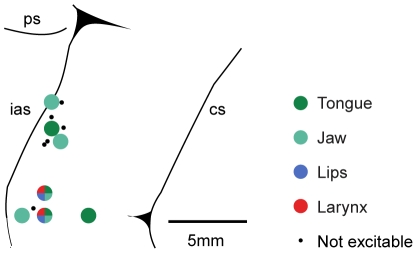
Representation of the penetrations in PMv in which long-train microstimulation evoked larynx, mouth and tongue movements. Other conventions as in Fig. 2.

We also applied long-train electrical microstimulation to the sites where vocalization-related neurons were found (see Materials and Methods); this enabled us to directly verify whether vocalization-selective neurons could produce a motor output. Out of 71 stimulated sites (19 penetrations), 29 were found to be excitable and elicited mouth movements. More specifically, they consisted mainly in jaw opening (38%) and closing (10%), tongue protrusion (28%) and retraction (14%) and lips retraction (31%) and protrusion (10%). These movements were often obtained in combinations. In 24% of excitable sites it was possible to elicit larynx cartilage movements together with tongue, jaw and lips movements (Figure 4).

## Discussion

### Behavioral results

Our behavioral data yield further confirmation that macaque monkeys can achieve a significant level of vocal control and learn to voluntarily emit vocalization when submitted to a vocal operant conditioning task [12], [13], [14]. While this type of task has been successfully used to increase rates of vocal response in various animal class or species like cats [38], dogs [39], rats [40] and birds [41], it is notoriously hard to achieve such results in nonhuman primates (see [6], [15]). Indeed, previous studies reported that vocalization performance in macaque can remain low even after a long over-training period [13], [16] and that it is very sensitive to context changes [6], [15].

One of the most critical of our behavioral findings is the occurrence of SV, a behavior emerging during training in both monkeys. This dissociation between mouth and larynx displayed during the vocalization task is not surprising. While it has been shown that monkeys can be successfully conditioned to make oro-facial actions like tongue protrusion [42], [43], jaw movements [18], [42], [44], and lips protrusion [45], the literature about vocal training suggests a limited degree of vocal control (see [15]).

Summing up, our results indicate that the larynx – at least for the scope of emitting vocalization – can be brought under partial voluntary control; and interestingly, the overall performance of our monkeys clearly reveal a dissociation between mouth and larynx control. This suggests that the cortical neural substrates responsible of the control of mouth and larynx for the purpose of voluntary vocalization, as opposed to those involved in emotional calls, do not reach a full coordination in the macaque monkey.

### Neurophysiological results

The main neurophysiological finding of the present study is that there are neurons active during conditioned vocalization in the lateral part of PMv. This same cortical sector codes also mouth motor acts such as licking, sucking and biting. This could raise the criticism that these discharges are due to mouth movements. However, our data indicate that, in order to obtain a vocalization-related neuronal response in PMv, the complete vocalization action - i.e. vocal fold contraction in coordination with movements of the articulators - leading to sound emission is required. Accordingly, vocalization-selective neurons do not respond during SV. In addition, the finding that no vocalization-selective neuron responds to air blowing excludes their possible involvement in controlling expiratory acts *per se*.

The specificity for conditioned vocalization shown by some PMv neurons is further supported by their different discharge in the two conditions (“Food”, “No Food”). One could argue that the vocalization-related discharge in the “Food” condition can be explained in terms of task outcome and/or reward expectation. Although some neurons in premotor cortex have been described as potentially modulated by a reward [46], [47], [48], [49], this interpretation is not very likely, due to the wide spectrum of timing (anticipatory or time-locked) and pattern of the discharge of vocalization-related neurons. Moreover, at difference with the former studies, in the present work we did not record neuronal responses related to the reward cue presentation or to the achievement of the reward. The temporal pattern of vocalization-selective responses, that is always time-locked to vocalization, appears to be more compatible with the motor control of different phases of it. This is also in agreement with the general properties of the neurons of this area, which can code specific temporal aspects of motor acts [50].

We hypothesize that the neuronal discharge difference in the “Food” and “No Food” conditions relies on the voluntary nature of “Food” vocalizations, that can be also revealed by their sound structure. In fact, we found a difference in F0, but not in F1 and F2, between conditioned and spontaneous vocalizations, thus indicating that in the former there is a lower tension on the vocal folds in absence of differences in the articulators. This suggests that in conditioned vocalization, PMv can easily control the articulatory component, but does not completely coordinate it with the vocal folds and expiratory components, as it would be predicted by the widely-accepted source-filter theory of speech production [51], [52], [53]. This hypothesis is also corroborated by the high frequency of SVs, in which only the articulatory component is present.

In a previous electrophysiological study carried out in the cingulate cortex [17], vocalization-related neurons have been found, intermingled with neurons coding oro-facial movements. Our findings show that a similar intermingling of these two groups of neurons also occurs in PMv. However, concerning vocalization-related neurons, there are two important differences between these two regions. First, most of the anticipatory units recorded in PMv maintained their discharge during sound emission, while those of the cingulate cortex stopped their firing at sound onset. Second, while all PMv neurons are excitatory, more than half of cingulate neurons show inhibitory responses. These differences could be explained by different roles exerted by cingulate and ventral premotor cortex in vocalization. The former would be involved in action initiation, while the latter would code the coordination of mouth articulatory acts and larynx movements aimed at sound production. According to this model, the cingulate cortex would play a “gating function” on the premotor cortical system [19]. In fact, lesion studies showed that monkeys with part of the anterior cingulate cortex removed are incapable to initiate conditioned vocalization, despite their preserved capacity to emit spontaneous calls [32].

The hypothesized role of PMv in coordinating mouth and larynx movements for the purpose of vocalization is evidenced, in the present work, by the elicitation, as a consequence of microstimulation, of associated movements of the larynx cartilage and those of the tongue and the mouth. The cortical sector in which these movements were evoked could partly correspond to those in which other authors [23], [54] described activation of larynx muscles in anaesthetized monkeys. In addition to the findings of these works, in the present study we provided a critical information: the PMv excitable sites evoking larynx movements match the presence of vocalization-related neurons. Although microstimulation experiments indicated the possibility of a larynx motor control from PMv, neuroanatomical experiments did not identify direct connection of the lateral sector of PMv with nucleus ambiguus. This nucleus is known to be directly involved in intrinsic and extrinsic laringeal muscles contraction, suggesting that the PMv control of phonation could occur indirectly through the reticular formation [54]. On the other hand, it is known that the lateral part of PMv has a direct control on the articulators (direct connection with the facial motor nucleus, [55]). In addition, low-threshold electrical stimulation of the lateral part of motor cortex elicits movements of the jaw, lips and tongue [56], [57], [58].

Altogether, these findings suggest that PMv vocalization neurons have a motor output on the subcortical structures that control vocalization acting on phonatory muscles and oro-facial articulators.

The motor control reported in the present paper is probably in part the result of vocal training. Although we cannot ascertain to which extent voluntary motor control can occur under natural conditions, it is likely that the neural machinery underlying it has a role in modifying the vocal tract and thus modulating the vocal output in untrained non-human primates. Behavioral studies [3], [4], [5] demonstrated that, in some species, the amplitude and duration of vocalizations are adapted to environmental parameters like background noise. It is possible that a basic voluntary vocal control is warranted for tuning vocalization according to contextual situations.

Previous studies showed that some PMv neurons are active not only when monkeys execute actions but also when they listen to the sound these actions produce (audio-visual mirror neurons, [59]). Furthermore, a PET study in macaques indicates that PMv could be involved in auditory processing of monkey calls [60]. In contrast with these findings, in vocalization-related neurons we did not find any specific response during listening to monkey vocalizations. This lack of acoustic properties suggests either that other types of neurons located in a different subregion of PMv are endowed with these properties or that motor and acoustic properties specific for vocalization are not yet fully coupled in the monkey PMv. Interestingly, other studies clearly showed that in the superior temporal and prefrontal cortices of the macaque there are neurons coding listened species-specific vocalizations [61], [62], [63]. While the possible motor properties of these neurons during vocalization were not investigated in macaques, two recent studies [64], [65], found that a sector of the prefrontal cortex of common marmoset increased early gene (cFOS and Egr-1) expression during antiphonal calling (i.e. a vocal response to a conspecific vocalization) but not during mere vocalization hearing. However, it is not clear whether these results reflect a motor component related to vocalization production, the decision making involved in the act of replying to another monkey or the retrieval of the appropriate vocal pattern. Altogether, these findings indicate that in monkeys the acoustic input reaching frontal areas might not be coupled with the motor representation of vocalizations, at difference with what occurs in other species, such as humans and songbirds [66], [67], [68], in which several aspects of the species-specific vocalization are learned through listening and practice.

### Conclusions

Several authors proposed, based on anatomical and functional evidence, that different parts of monkey PMv can be homologue of human area 44 [25], [69], [70]. The location in the lateral part of area F5 of the vocalization-related neurons recorded in the present study supports this homology. This would imply that this region, beyond controlling the oro-facial movements involved in ingestive and communicative gestures [71], could combine this control with that of sound emission. The identification of neurons coding voluntary vocalization favors the idea that this type of coding could be a precursor of a volitional phonatory-based communicative system that dramatically expanded in the human lineage.

## Supporting Information

Figure S1 **Lips configuration during coo-calls and SVs.** Three examples of sequences of coos and SVs are shown. Each example was taken during the same recording session. Note that coos and SVs involve similar mouth configuration and timing in their unfolding. The third frame of each sequence corresponds to the maximum lips protrusion.
(TIF)

Figure S2 **Neuronal activity and sound features related to different behavioral events during “Food” condition.** Top panel. Coo and SV; Bottom panel. Coo and Air blow. Note that vocalization-related neurons do not fire during the emission of SV or Air blow.
(TIF)

Movie S1 **Example of coo-call.**
(MOV)

Movie S2 **Example of SV.**
(MOV)
