# Supplementary figures and images for: Neurons Controlling Voluntary Vocalization in the Macaque Ventral Premotor Cortex
Source: PLoS One. 2011 Nov 2;6(11):e26822. doi: 10.1371/journal.pone.0026822 (PMC3206851; doi:10.1371/journal.pone.0026822)

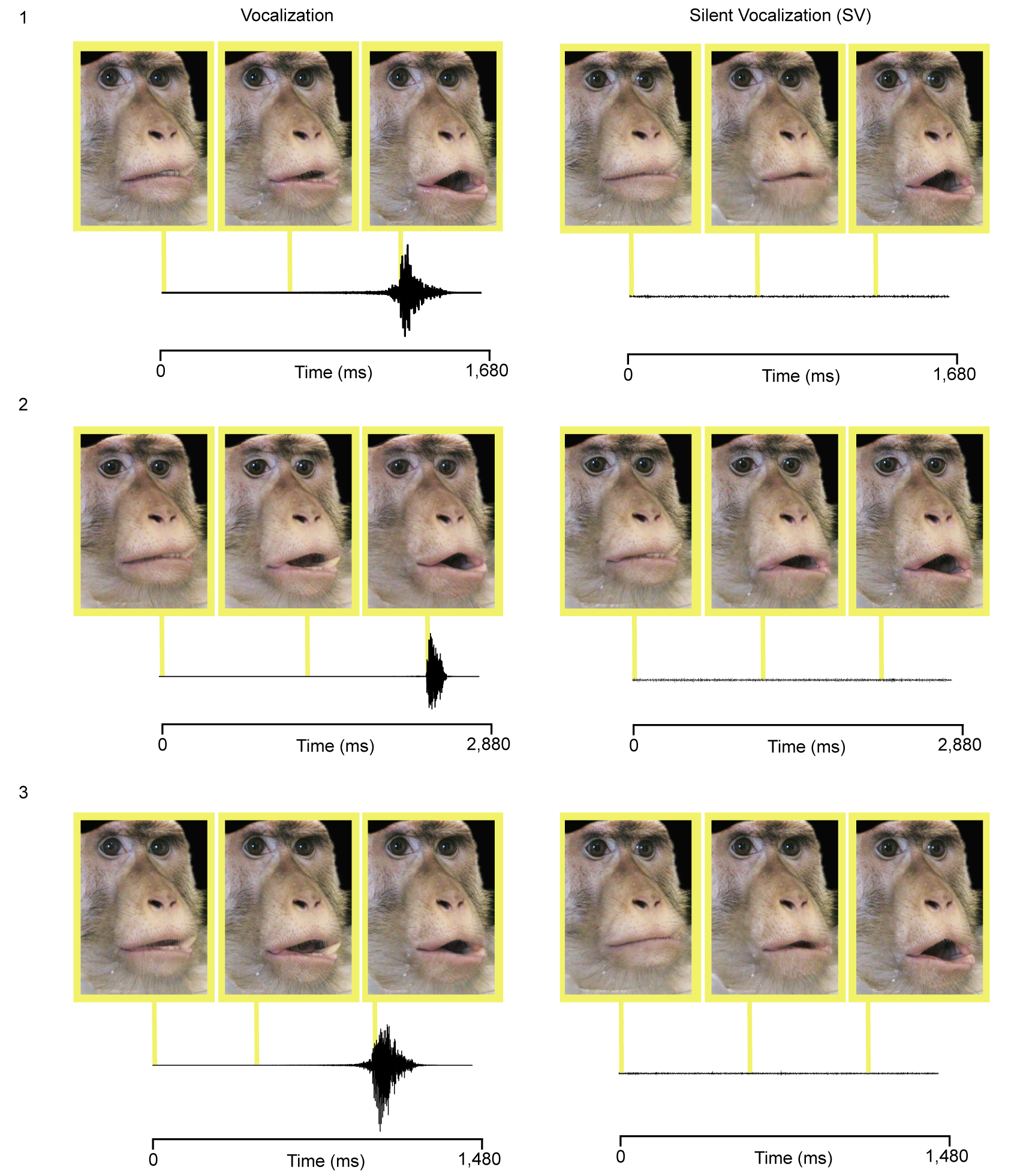

Supplement: Figure S1 — Lips configuration during coo-calls and SVs. Three examples of sequences of coos and SVs are shown. Each example was taken during the same recording session. Note that coos and SVs involve similar mouth configuration and timing in their unfolding. The third frame of each sequence corresponds to the maximum lips protrusion. (TIF) [file pone.0026822.s001.tif]

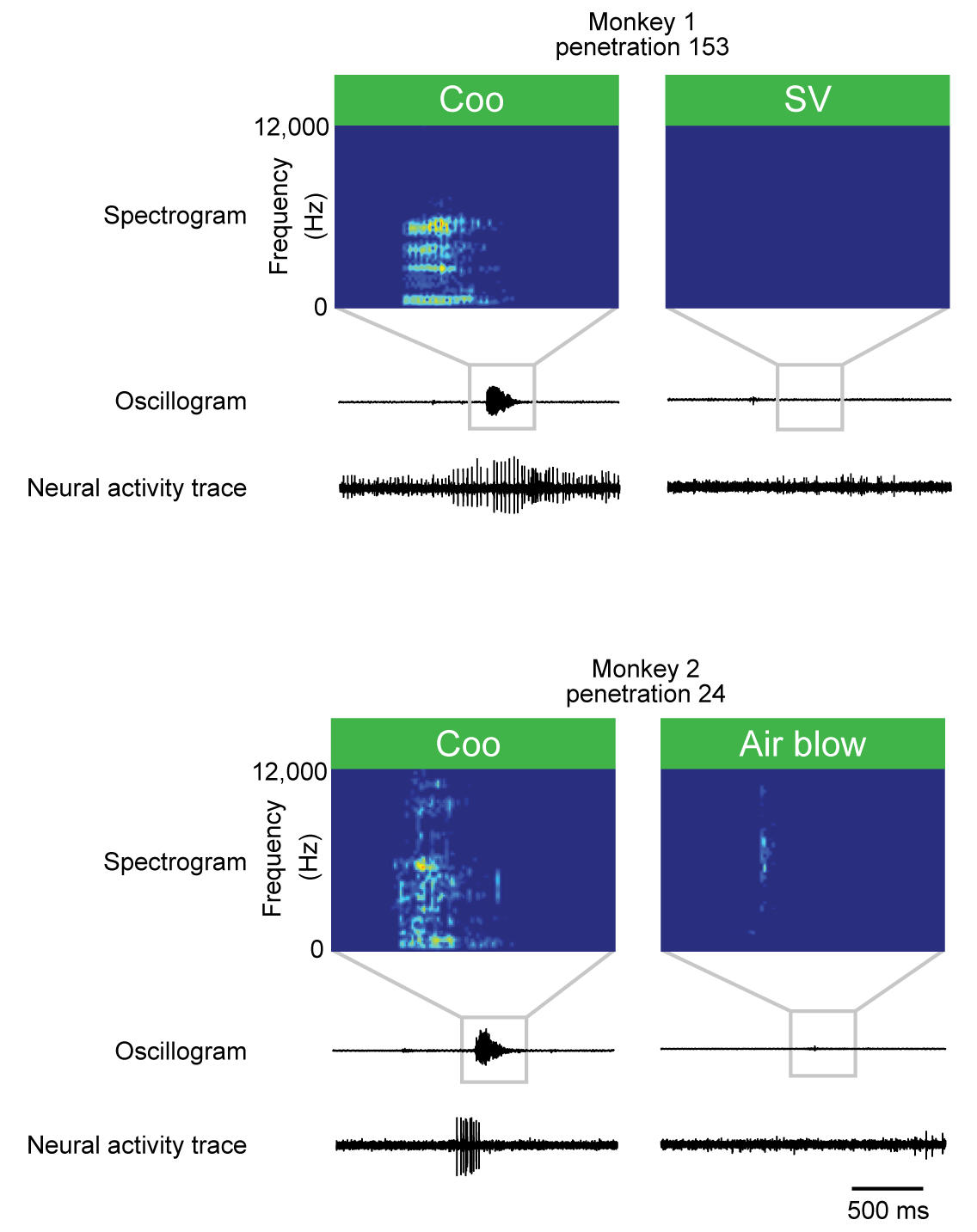

Supplement: Figure S2 — Neuronal activity and sound features related to different behavioral events during “Food” condition. Top panel. Coo and SV; Bottom panel. Coo and Air blow. Note that vocalization-related neurons do not fire during the emission of SV or Air blow. (TIF) [file pone.0026822.s002.tif]
